# Heregulin expression and its clinical implication for patients with *EGFR*-mutant non-small cell lung cancer treated with EGFR-tyrosine kinase inhibitors

**DOI:** 10.1038/s41598-019-55939-5

**Published:** 2019-12-20

**Authors:** Kimio Yonesaka, Eiji Iwama, Hidetoshi Hayashi, Shinichiro Suzuki, Ryoji Kato, Satomi Watanabe, Takayuki Takahama, Junko Tanizaki, Kaoru Tanaka, Masayuki Takeda, Kazuko Sakai, Koichi Azuma, Yasutaka Chiba, Shinji Atagi, Kazuto Nishio, Isamu Okamoto, Kazuhiko Nakagawa

**Affiliations:** 1 Department of Medical Oncology, Kindai University Faculty of Medicine, Osaka-sayama, Osaka, 589-8511 Japan; 2 Research Institute for Disease of the Chest, Kyushu University Faculty of Medicine, Fukuoka City, Fukuoka, 812-8582 Japan; 3 Department of Genome Biology, Kindai University Faculty of Medicine, Osaka-sayama, Osaka, 589-8511 Japan; 4 Division of Respirology, Neurology, and Rheumatology, Department of Internal Medicine, Kurume University School of Medicine, Kurume, Fukuoka, 830-0011 Japan; 5 Clinical Research Center, Kindai University Hospital, Osaka-sayama, Osaka, 589-8511 Japan; 6 Department of Thoracic Oncology, Kinki-chuo Respiratory Medical Center, Sakai, Osaka, 591-8555 Japan

**Keywords:** Non-small-cell lung cancer, Tumour biomarkers

## Abstract

Epidermal growth factor receptor-tyrosine kinase inhibitors (EGFR-TKIs) are standard therapy for *EGFR*-mutant non-small cell lung cancer (NSCLC). Preclinically, HER3 ligand heregulin induces resistance to EGFR-TKIs, whereas the pan-human EGFR family inhibitor afatinib remains effective. Here, we examined whether soluble heregulin levels have clinical implications for *EGFR*-mutant NSCLC treated with EGFR-TKIs. Soluble heregulin was immunologically measured in plasma from *EGFR*-mutant NSCLC patients. Cutoff values were determined by 1-year PFS ROC curve. The relationship between soluble heregulin and PFS following EGFR-TKI therapy was analyzed by Cox proportional hazards model. Seventy-three patients were enrolled: 44 were treated with 1^st^-generation and 29 with 2^nd^-generation EGFR-TKIs. Soluble heregulin levels varied (range: 274–7,138 pg/mL, median: 739 pg/mL). Among patients treated with 1^st^-generation EGFR-TKIs, those with high heregulin (n = 20, >800 pg/mL) had a tendency for shorter PFS than those with low heregulin (n = 24, <800 pg/mL), with median PFS of 322 and 671 days, respectively. Cox proportional hazards model also indicated a trend toward resistance against 1^st^-generation EGFR-TKIs (HR: 1.825, 95% CI: 0.865–4.318) but not against 2^nd^-generation EGFR-TKIs. Soluble heregulin potentially correlates with resistance to EGFR-TKIs but not 2^nd^-generation EGFR-TKIs in patients with *EGFR*-mutant NSCLC.

## Introduction

Epidermal growth factor receptor (EGFR) is a critical molecular target of anti-cancer therapy in non-small cell lung cancer (NSCLC)^1,2^. Previous clinical trials have demonstrated that EGFR tyrosine kinase inhibitors (EGFR-TKIs), such as gefitinib and erlotinib, dramatically improve the survival of patients with NSCLC harboring EGFR-activating mutations^3–5^. EGFR genomic mutations alter its protein structure, at the site where ATP preferentially binds to its intracellular kinase domain, leading to spontaneous EGFR activation^6,7^. However, some tumors are refractory to EGFR-TKI therapy despite harboring EGFR-activating mutations. Even though tumors respond to EGFR-TKI therapy, they eventually become resistant to it. Several underlying mechanisms of resistance to EGFR inhibitors have already been identified^8^. In particular, the T790M secondary *EGFR* mutation was detected in approximately 50% of the patients with NSCLC harboring EGFR-activating mutations with acquired resistance to EGFR-TKIs^9^. Furthermore, other resistant mechanisms, including *MET* amplification, human EGFR 2 (*HER2*) amplification, and hepatocyte growth factor overexpression, have also been reported in NSCLC^10–12^. Intriguingly, MET aberrant expression leads to activation of HER3 and its downstream pathway, suggesting that HER3 plays a key role in EGFR-TKI resistance^10^. Based on these findings, it is imperative that novel treatment strategies are clinically investigated to overcome EGFR-TKI resistance.

Second- or third-generation EGFR-TKIs demonstrate superior clinical efficacy for EGFR-TKI-naïve patients compared to that of 1^st^-generation EGFR-TKI^13–15^. 2^nd^-generation EGFR-TKIs, including afatinib and dacomitinib, can irreversibly bind to EGFR and other HER family tyrosine kinases and thus are referred to as pan-HER inhibitors^16,17^. In randomized clinical trials, second-generation EGFR-TKIs were shown to significantly improve progression-free survival (PFS) as well as overall survival compared to first-generation EGFR-TKIs in patients with advanced NSCLC harboring EGFR-activating mutations^13,14^. However, second-generation EGFR-TKIs were not able to overcome EGFR T790M-induced resistance to EGFR-TKIs^18^. In fact, like those treated with first-generation EGFR-TKIs, the secondary EGFR T790M mutation is present in approximately 50% of patients treated with second-generation EGFR-TKIs^19^. In contrast to first- and second-generation EGFR-TKIs, third-generation EGFR-TKIs, such as osimertinib, have been shown to exhibit enhanced efficacy against NSCLC with the EGFR T790M mutation^20,21^. Furthermore, osimertinib significantly improves PFS in EGFR-TKI-naïve patients with NSCLC harboring EGFR-activating mutations compared to first-generation EGFR-TKIs^15^. However, despite pharmacodynamic improvements in EGFR-TKIs, a subset of patients with NSCLC harboring EGFR-activating mutations continue to exhibit resistance to EGFR-TKIs.

Heregulin is a ligand for HER3 and HER4 and is aberrantly overexpressed in cancer cells, including NSCLC or cancer-associated fibroblast cells^22–24^. Heregulin alters the conformational structure of its binding receptors and may activate HER3, HER4, and its coupling partner HER2 in cancer cells in an autocrine or paracrine manner^25,26^. Previously, preclinical studies have suggested that heregulin may cause resistance to first-generation EGFR-TKIs such as erlotinib in NSCLC patients harboring EGFR-activating mutations, as heregulin promotes HER2-HER3 coupling and activates anti-apoptotic HER2-HER3-Akt bypass signaling^27^. In contrast to 1^st^-generation EGFR-TKIs, 2nd-generation EGFR-TKIs such as afatinib or dacomitinib, unique pan-HER family inhibitors, have been shown preclinically to overcome heregulin-mediated resistance^28^. Heregulin expression varies in patients with NSCLC harboring EGFR-activating mutations, although its clinical implications are unclear, especially in terms of EGFR-TKI therapeutic efficacy^28^.

In the current study, we aimed to exploratively examine whether the soluble heregulin (sHRG) level in plasma has clinical implications for EGFR-TKI efficacy in patients with NSCLC harboring EGFR-activating mutations. First- and second-generation of EGFR-TKIs were assessed to determine their efficacy in patients with high heregulin expression.

## Results

### Patient characteristics and cutoff values for sHRG

**Figure 1:**
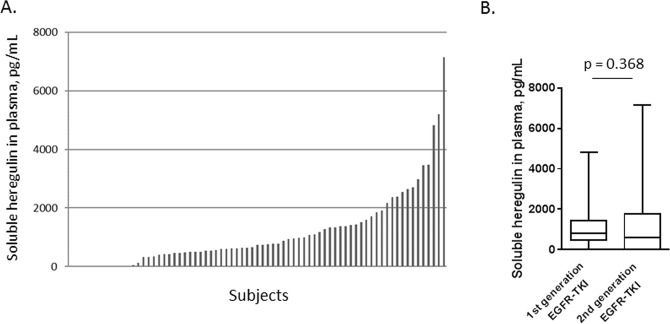
(**A**) Soluble heregulin expression in patients with NSCLC with EGFR-activating mutations. Soluble heregulin was measured in plasma obtained from patients prior to EGFR-TKI treatment by quantitative sandwich immune assay (n = 76). X-axis, individual patients; y-axis, plasma heregulin concentration, pg/mL. (**B**) Boxplot shows soluble heregulin expression for patients on 1^st^ and 2^nd^ generation EGFR-TKI. The Mann-Whitney test was used to compare differences between patients on 1^st^ and 2^nd^ generation EGFR-TKI.

A total of 73 patients with NSCLC harboring EGFR-activating mutations were enrolled in this study. All patients had been treated with EGFR-TKIs between February 2015 and July 2018. Of those patients, 44 patients received first-generation EGFR-TKIs (gefitinib or erlotinib), and 29 patients received second-generation EGFR-TKIs (afatinib or dacomitinib). Plasma samples had been collected prior to EGFR-TKI therapy in all 73 patients and was used for measuring sHRG protein levels. The sHRG distribution in these patients is shown in Fig. 1A. sHRG levels varied, ranging from 274 pg/mL (the lower limit of detection) to 7,138 pg/mL, with a median concentration of 739 pg/mL. Patients treated with first- or second-generation EGFR-TKIs were grouped together for analysis. The sHRG levels did not significantly differ between those groups (Fig. 1B). Baseline characteristics for all patients, as well as for the 1-generation EGFR-TKI population and the 2^nd^-generation EGFR-TKI population are shown in Table 1. Characteristics were similar between these two subpopulations, although those receiving second-generation EGFR-TKI therapy more frequently included smokers and patients with minor *EGFR* mutations, such as exon 20 insertion and exon 18 point mutations G719S, G719A, and G719C. Those characteristics did not significantly correlate with sHRG levels (Supplemental Fig. 1). The median PFS for the 1^st^-generation EGFR-TKI population and 2^nd^-generation EGFR-TKI population were 446 and 393 days, respectively, and a survival curve for each population is shown in Supplemental Fig. 2.

**Table 1:** Patient characteristics.

	All patients (n = 73)	Treatment with 1^st^-generation EGFR-TKIs	Treatment with 2^nd^-generation EGFR-TKIs
		(n = 44)	(n = 29)
Age, median years (range)	71 (37–91)	70.5 (44–91)	71 (37–82)
<70	34 (46.6)	21 (47.7)	13 (44.8)
>70	39 (53.4)	23 (52.3)	16 (55.2)
**Sex, n (%)**			
Male	34 (46.6)	18 (40.9)	16 (55.2)
Female	39 (53.4)	26 (59.1)	13 (44.8)
**Smoking status, n (%)**			
Never	40 (54.8)	28 (63.6)	12 (41.4)
Smoker	33 (45.2)	16 (36.4)	17 (58.6)
**Tumor histology subtype, n (%)**			
Adenocarcinoma	71 (97.3)	43 (97.7)	28 (96.6)
Large cell carcinoma	0 (0)	0 (0)	0 (0)
Other	2 (2.7)	1 (2.3)	1 (3.4)
**EGFR mutation status, n (%)**			
Exon 19 deletion	29 (39.7)	16 (36.4)	13 (44.8)
Exon 21 L858R	38 (52.1)	28 (63.6)	10 (34.5)
Other	6 (8.2)	-	6 (20.7)
**ECOG performance status, n (%)**			
0	15 (20.6)	8 (18.2)	7 (24.1)
1	51 (69.9)	29 (65.9)	22 (75.9)
2	5 (6.8)	5 (11.4)	0 (0)
3, 4	2 (2.7)	2 (4.5)	0 (0)
**Number of prior NSCLC therapies, n (%)**			
0	64 (87.7)	39 (88.6)	25 (86.2)
1	9 (12.3)	5 (11.4)	4 (13.8)
**Clinical stage, n (%)**			
Post-operative or radiative relapse	5 (6.8)	0 (0)	5 (17.2)
3B	0 (0)	0 (0)	0 (0)
4	68 (93.2)	44 (100)	24 (82.8)
**Type of EGFR-TKI, n (%)**			
1^st^-generation (erlotinib, gefitinib)	44 (60.3)	44 (100)	0 (0)
2^nd^-generation (afatinib, dacomitinib)	29 (39.7)	0 (0)	29 (100)

EGFR-TKI = epidermal growth factor receptor tyrosine kinase inhibitor, NSCLC = non-small cell lung cancer.

For subsequent analysis of the 1^st^-generation EGFR-TKI population, 1-year PFS ROC curve analysis was performed for determining the cutoff values for classifying patients into sHRG-high and sHRG-low groups. The optimal cutoff value was located between 787 and 884 pg/mL; therefore a cutoff value of 800 pg/mL was selected, and 25 patients were classified into the sHRG-low group, with 22 patients classified into the sHRG-high group (Supplemental Fig. 3).

### PFS in the 1^st^-generation EGFR-TKI population

**Figure 2:**
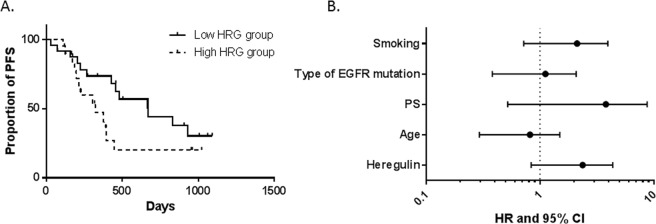
Kaplan–Meier curves of progression-free survival in the 1^st^-generation EGF-TKI population. (**A**) Kaplan–Meier survival curve was drawn for patients classified as sHRG-high (n = 20) and sHRG-low (n = 24). (**B**) Cox proportional hazards model adjusted by factors including smoking, type of *EGFR* mutation, performance status, age, and heregulin expression.

For the 1^st^-generation EGFR-TKI population, treatment efficacy as measured by PFS was shorter in the sHRG-high subgroup than in the sHRG-low subgroup (Fig. 2A). The median PFS of the sHRG-high and sHRG-low subgroups were 322 days and 671 days, respectively [hazard ratio (HR): 1.825; 95% CI: 0.865–4.318; log-rank test p-value = 0.1137]. Furthermore, Cox proportional hazards analysis for PFS showed that the sHRG-high subgroup tended to exhibit resistance to EGFR-TKI treatment, after correcting for several factors including age, performance status, type of *EGFR* mutation, and smoking (HR: 1.911; 95% CI: 0.837–4.360; p-value = 0.124, Fig. 2B).

### PFS in the second-generation EGFR-TKI population

**Figure 3:**
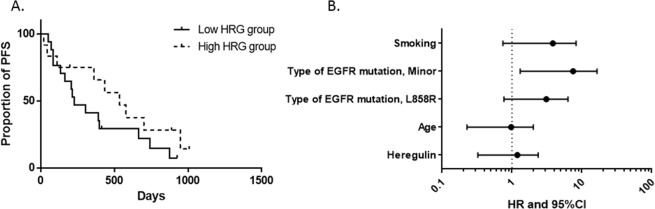
Kaplan–Meier curves of progression-free survival in 2^nd^-generation EGF-TKI population. (**A**) Kaplan-Meier survival curve was drawn for patients classified as sHRG-high (n = 12) and sHRG-low (n = 17). (**B**) Cox proportional hazards model adjusted by factors including smoking, type of *EGFR* mutation, age, and heregulin expression.

Subsequently, we examined whether resistance to second-generation EGFR-TKIs was similarly related to sHRG levels, as observed in the first-generation EGFR-TKI population. Twenty-nine patients were classified into sHRG-low (n = 17) and sHRG-high subgroups (n = 12) using the same cutoff value of 800 pg/mL as determined for the 1^st^-generation EGFR-TKI population. In contrast to the results for the first-generation EGFR-TKI population, the efficacy of second-generation EGFR-TKIs was more durable in the sHRG-high subgroup than in the sHRG-low subgroup (Fig. 3A). The median PFS of the sHRG-high and sHRG-low subgroups were 535 days and 228 days, respectively (HR: 0.5978; 95% CI: 0.262–1.298; log-rank test p-value = 0.2019). However, it should be noted that patients with minor *EGFR* mutations were frequently included in the sHRG-low subgroup. Cox proportional hazards regression analysis for PFS indicated that in the sHRG-high group, there was no obvious correlation between sHRG expression and EGFR-TKI resistance, after correcting for several factors including age, type of *EGFR* mutation, and smoking (HR: 0.879; 95% CI: 0.325–2.376; p-value = 0.799, Fig. 3B).

## Discussion

In this study, we observed the potential implications of heregulin expression in EGFR-TKI–treated NSCLC patients who harbored EGFR-activating mutations. The efficacy of 1st-generation EGFR-TKIs was less durable in patients with high sHRG plasma levels than in patients with low sHRG plasma levels. Furthermore, Cox regression analysis showed that this tendency was maintained after adjusting for multiple influential factors such as PS, smoking history, and age^29^. This study generated a new hypothesis, which states that soluble heregulin levels might be associated with the limited efficacy of EGFR-TKIs in NSCLC patients who harbor EGFR-activating mutations.

This study could not confirm the statistical significance of the association between heregulin plasma levels and limitations in the efficacy of EGFR-TKIs. Moreover, the hazard ratio for PFS crossed 1.0 in the 1^st^-generation subgroup of EGFR-TKI patients. Our previous preclinical study suggested that heregulin expression causes EGFR-TKI resistance in EGFR-mutant NSCLC^27^. However, the degree of heregulin influence in clinical situations remains unknown. Thus, the optimal cutoff point for high heregulin expression levels could not be determined. For those reasons, we could not statistically determine appropriate sample sizes prior to this study. A subsequent study is warranted for validating our new hypothesis with statistically appropriate sample sizes in order to optimize EGFR-TKI therapy in patients with EGFR-mutant NSCLC.

Recently, the 3^rd^-generation EGFR-TKI osimertinib was shown to significantly improve PFS and overall survival rates in EGFR-TKI–naive patients compared to 1^st^ generation EGFR-TKIs^15,30^. However, a preclinical study demonstrated that heregulin-expressing NSCLC cells are resistant to osimertinib (Supplement Fig. 4). Considering those results, the implications of heregulin expression should be investigated in osimertinib-treated patients with EGFR-mutant NSCLC.

This study is the first to report the clinical implications of heregulin expression in EGFR-TKI–treated NSCLC patients harboring EGFR-activating mutations; the observation indicates a prognostically unfavorable influence of heregulin. However, in the other cohort, we did not find any prognostic influence of heregulin in NSCLC (Supplemental Fig. 5). Thus, although this study had a limited sample size, we speculate that sHRG levels may predict resistance to EGFR-TKIs in this population. A previous preclinical study found that induced expression of the heregulin gene worsened the sensitivity to the EGFR-TKI erlotinib of an NSCLC cell line harboring an EGFR-activating mutation^27^. Although the current study did not evaluate heregulin expression levels in tumors, sHRG found in plasma may be potentially produced in tumors and may limit the efficacy of EGFR-TKIs. The current clinical observations have not yet been validated in other cohorts. However, similar to the findings of the current study, patients with advanced colorectal cancer with high plasma sHRG levels exhibited shorter PFS following anti-EGFR antibody therapy than those with low sHRG^31^. Considering these observations, sHRG expression may potentially be associated with resistance to EGFR-TKIs, regardless of cancer type, and confirmation of this relationship in an unbiased study is warranted.

In contrast to 1^st^-generation EGFR-TKIs, 2^nd^-generation EGFR-TKIs showed no obvious relationship between heregulin expression and EGFR-TKI resistance according to the Cox proportional hazards model. Although the sample size was small, this difference between the generations of EGFR-TKIs may be caused by differences in pharmacological action. Specifically, the anti-cancer effects of second-generation EGFR-TKIs on heregulin-expressing cancer cells by be sustained by pan-HER family inhibition. In fact, a preclinical study demonstrated that the second-generation EGFR-TKI afatinib uniquely decreased EGFR activation, as well as that of HER2, HER3, HER4, and their downstream Akt phosphorylation, in heregulin-expressing cancer cells, overcoming heregulin-mediated resistance^28^. Furthermore, second-generation EGFR-TKIs have demonstrated a superior survival benefit compared to that of first-generation EGFR-TKIs in randomized clinical trials, and the mechanism of this effect was considered to be an advantage in pan-HER family inhibition specific to second-generation EGFR-TKIs^13,14^. Although other mechanisms, such as a *HER2* genomic amplification, may activate HER family members other than EGFR, heregulin may potentially play a critical role in HER2, 3, and 4 activation. Considering this, NSCLC patients with heregulin expression may be an optimal subpopulation for second-generation EGFR-TKI treatment.

The current study did not examine whether sHRG levels were correlated with tumor heregulin expression. However, we previously examined this relationship in another cohort of NSCLC patients but did not observe a significant correlation^32^. This may imply heterogeneous heregulin expression levels among tumors. Alternatively, the sHRG level in the plasma may be influenced by the tumor burden, such as the size of the tumor or the number of metastases. Moreover, the measurement of sHRG for evaluating the local heregulin expression in tumors may be technically limited, whereas the current results imply that sHRG levels may be advantageous for evaluating systemic heregulin expression and may by proxy reflect resistance to EGFR-TKIs.

In conclusion, results of the current study suggest the potential clinical implications of heregulin expression in EGFR-TKI treatment–naive NSCLC patients with EGFR-activating mutations. Specifically, sHRG levels potentially correlate with resistance to first-generation EGFR-TKIs, but not to second-generation EGFR-TKIs capable of pan-HER family inhibition.

## Methods

### Study design

This was a retrospective cohort study. Patients were eligible for enrollment in the study if they had histologically confirmed NSCLC with *EGFR* mutation; had undergone stage IIIB/IV, post-operative, or radiation therapy; had measurable disease (per Response Evaluation Criteria in Solid Tumors guidelines, version 1.1); and had been treated with an EGFR-TKI without prior EGFR-TKI therapy^33^. EGFR-TKI re-challenge therapy or secondary EGFR-TKI therapy was not eligible. The following EGFR-TKIs were included: gefitinib, erlotinib, afatinib, and dacomitinib. The primary objective was to assess the correlation between the heregulin level and PFS in patients treated with each generation of EGFR-TKIs. The protocol was approved by the institutional review boards of the participating institutions including the Institutional Review Board of Kindai University Faculty of Medicine, Kyushu University Institutional Review Board for Clinical Research, the Institutional Review Board of Kurume University, and the Institutional Review Board of the Kinki-Chuo Respiratory Medical Center. Subjects provided written informed consent, including consent to provide plasma samples for assessment of heregulin expression. All methods including sHRG measurement were performed in accordance with Ethical Guidelines for Medical and Health Research Involving Human Subjects.

### Biomarker assay for sHRG

Plasma samples were obtained from participants prior to EGFR-TKI treatment. sHRG was measured using a validated quantitative sandwich immune assay using a commercially available kit (NRG1 beta 1 human ELISA Kit, Abcam, Cambridge, MA, USA) according to our modified method^28^. Specifically, a 96-well microplate coated with anti-NRG1-β1 capture antibody was incubated with samples and standards. The plate was washed, probed with anti-NRG1-β1 detection antibody, and labeled with a chromogen. Finally, the optical densities of samples and standards were determined using a spectrophotometric microplate reader at 450 nm. The sHRG concentration of each sample was determined based on standard curves.

### Statistical analyses

PFS was defined as the duration from the initiation of EGFR-TKI therapy until tumor progression or death from any cause. Kaplan–Meier curves were generated for PFS and used to calculate the median and 95% CI of each treatment group. Two-sided p-values were determined by log-rank test, and hazard ratios (and 95% CIs) were determined by the Cox proportional hazards model, stratified by age, smoking history, clinical stage, type of *EGFR* mutation, and Eastern Cooperative Oncology Group performance status. Analyses were performed using SPSS (version 22, SPSS Inc., Chicago, IL, USA). Data were graphically displayed using GraphPad Prism v.5.0 for Windows (GraphPad Software, Inc., La Jolla, CA, USA).

## Supplementary information

Supplementary Figures

## Data Availability

The datasets generated during the current study are available from the corresponding author on reasonable request.
