# Supplementary material for: Heregulin expression and its clinical implication for patients with EGFR-mutant non-small cell lung cancer treated with EGFR-tyrosine kinase inhibitors
Source: Sci Rep. 2019 Dec 20;9:19501. doi: 10.1038/s41598-019-55939-5 (PMC6925200; doi:10.1038/s41598-019-55939-5)
Supplement: Supplementary file 1 — Supplementary Figures [file 41598_2019_55939_MOESM1_ESM.pdf]

Title: Heregulin expression and its clinical implication for patients with *EGFR*-mutant non-small cell lung cancer treated with EGFR-tyrosine kinase inhibitors

Authors list: Kimio Yonesaka[1]*, Eiji Iwama[2], Hidetoshi Hayashi[1], Shinichiro Suzuki[1], Ryoji Kato[1], Satomi Watanabe[1], Takayuki Takahama[1], Junko Tanizaki[1], Kaoru Tanaka[1], Masayuki Takeda[1], Kazuko Sakai[3], Koichi Azuma[4], Yasutaka Chiba[5], Shinji Atagi[6], Kazuto Nishio[3], Isamu Okamoto[2], and Kazuhiko Nakagawa[1]

Affiliations list: [1]Department of Medical Oncology, Kindai University Faculty of Medicine, Osaka-sayama, Osaka 589-8511, Japan
[2]Research Institute for Disease of the Chest, Kyushu University Faculty of Medicine, Fukuoka City, Fukuoka 812-8582, Japan
[3]Department of Genome Biology, Kindai University Faculty of Medicine, Osaka-sayama, Osaka 589-8511, Japan
[4]Division of Respirology, Neurology, and Rheumatology, Department of Internal Medicine, Kurume University School of Medicine, Kurume, Fukuoka 830-0011, Japan
[5]Clinical Research Center, Kindai University Hospital, Osaka-sayama, Osaka 589-8511, Japan
[6]Department of Thoracic Oncology, Kinki-chuo Respiratory Medical Center, Sakai, Osaka 591-8555, Japan

*Corresponding Author: Kimio Yonesaka, Department of Medical Oncology, Kindai University Faculty of Medicine, 377-2 Ohno-higashi Osaka-Sayamashi, Osaka 589-8511, Japan. Phone: 81-72-366-0221; Fax: 81-72-360-5000; E-mail: yonesaka@med.kindai.ac.jp

Supplement Figure 1. Correlation between heregulin expression and characteristics

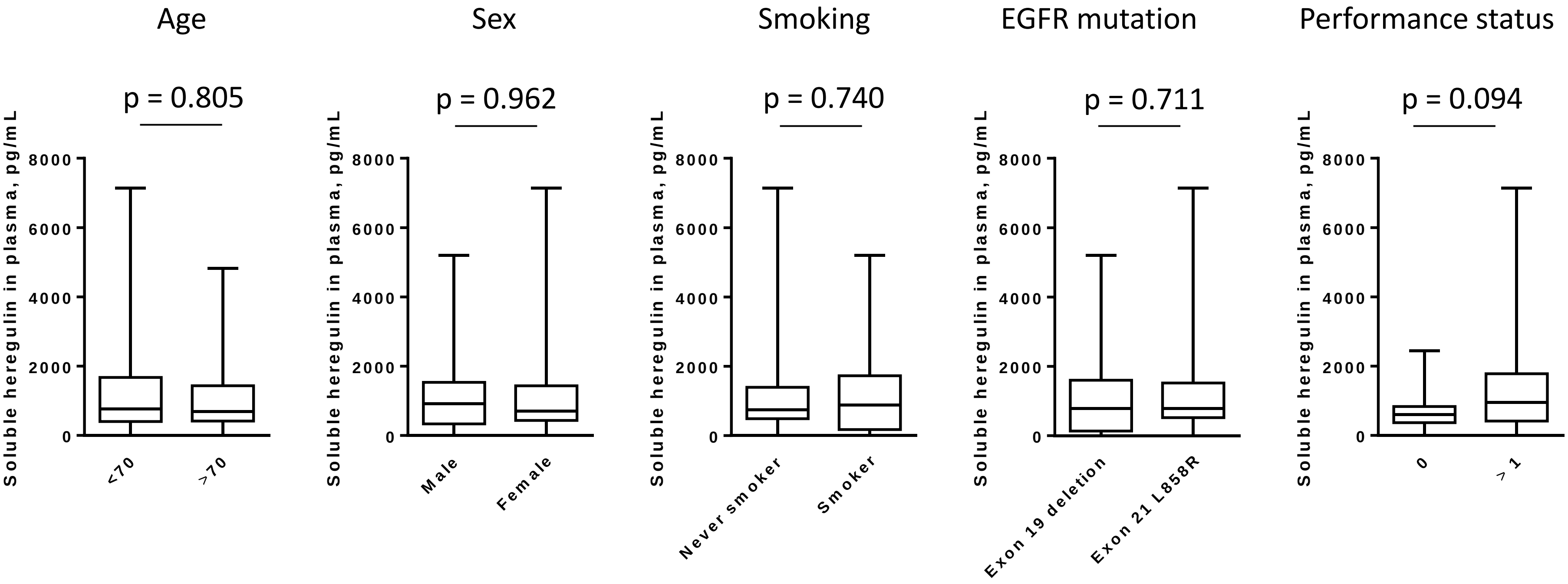

Boxplot shows soluble heregulin expression in each patient characteristic. X-axis, patient characteristic ; y-axis, plasma heregulin concentration, pg/mL. The Mann-Whitney test was performed for statistical analysis.

Supplement Figure 2. PFS survival curve of each TKIs

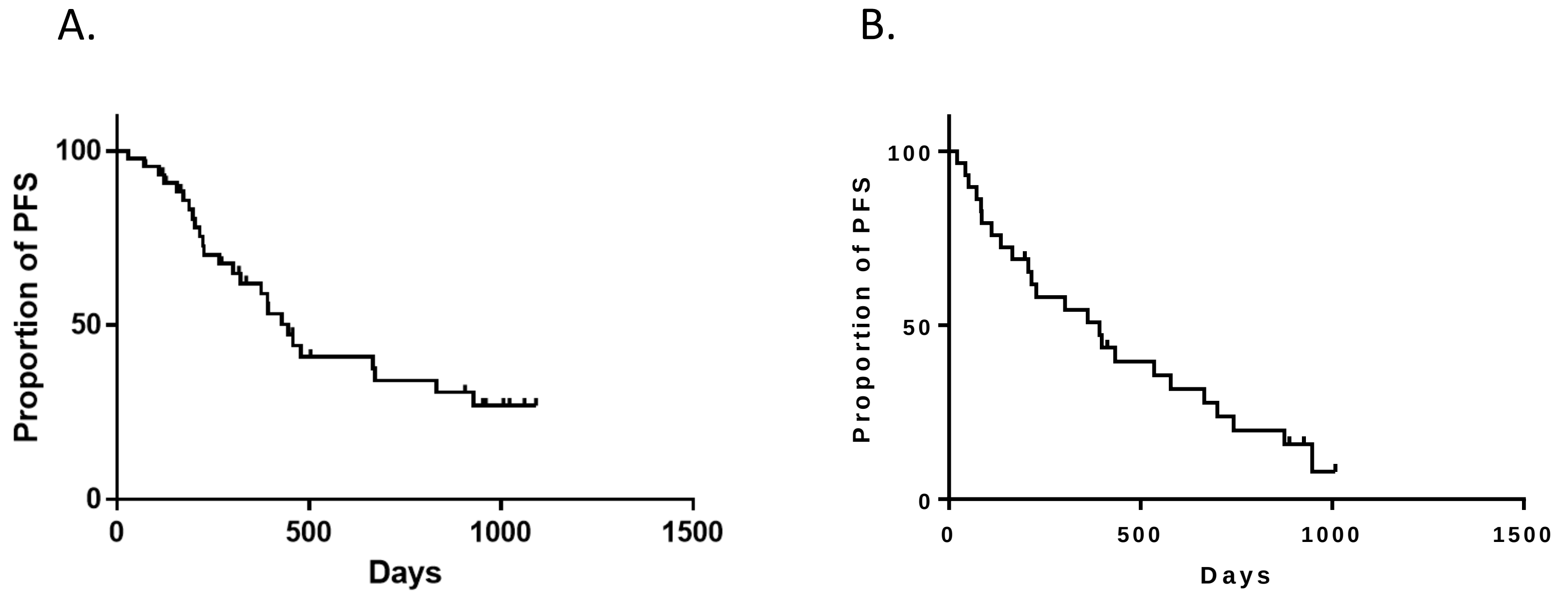

Kaplan-Meier curves of progression-free survival in 1st generation EGF-TKI population or 2nd generation EGFR-TKI population. A. Kaplan-Meier curve was drawn for patients treated with 1st generation EGFR-TKIs (n = 44). B. Kaplan-Meier curve was drawn for patients treated with 2nd generation EGFR-TKIs (n = 29).

Supplement Figure 3. ROC curve

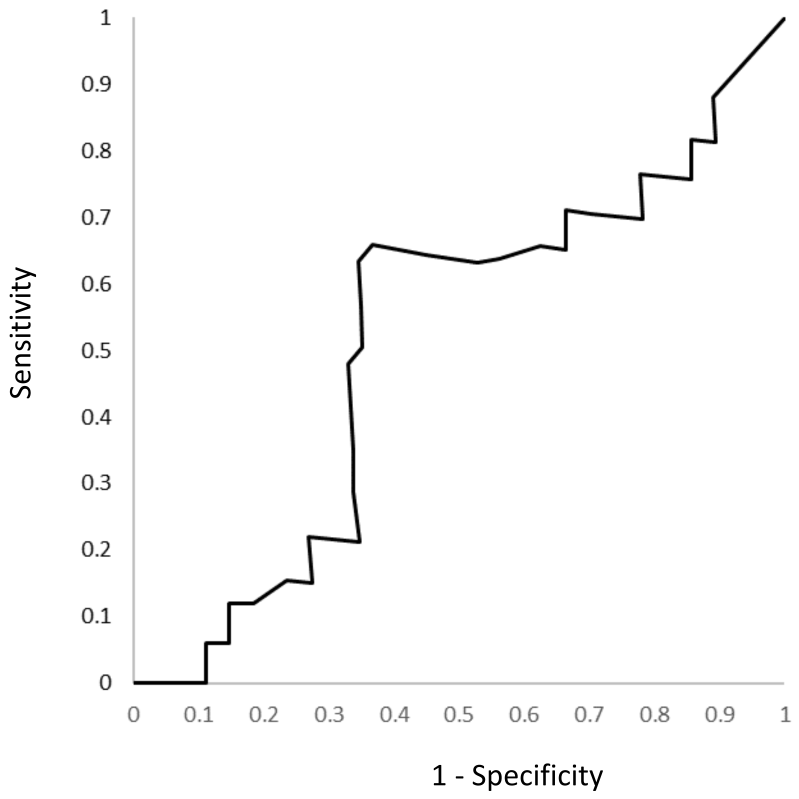

Receiver operating characteristics curves with optimal thresholds for PFS calculated from soluble heregulin concentration. Cuttoff values 787 mg/ml corresponding specificity / sensitivity (0.63 / 0.66) for predicting PFS. Area under the curve is 0.51.

Supplement Figure 4.

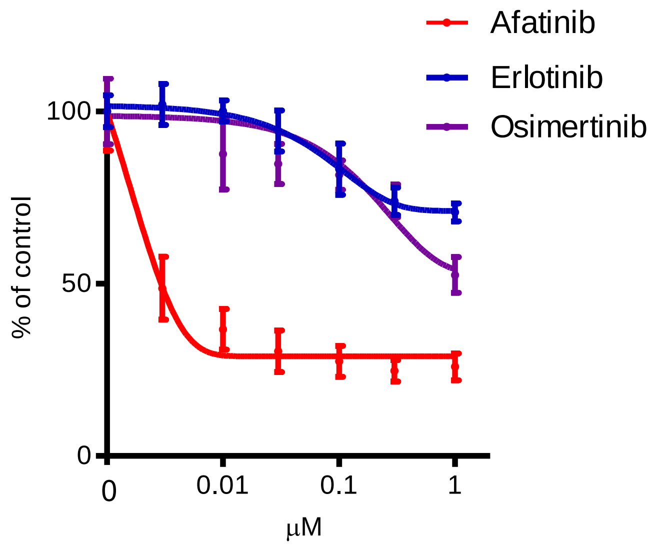

Heregulin expressing EGFR mutant non-small cell lung cancer PC9HRG cells were treated with the indicated concentrations of afatinib, erlotinib, or osimertinib and cell viability was measured 3 days later in vitro. Values are plotted relative to untreated control cells (n = 6, 95% confidence interval for the mean).

Supplement Figure 5.

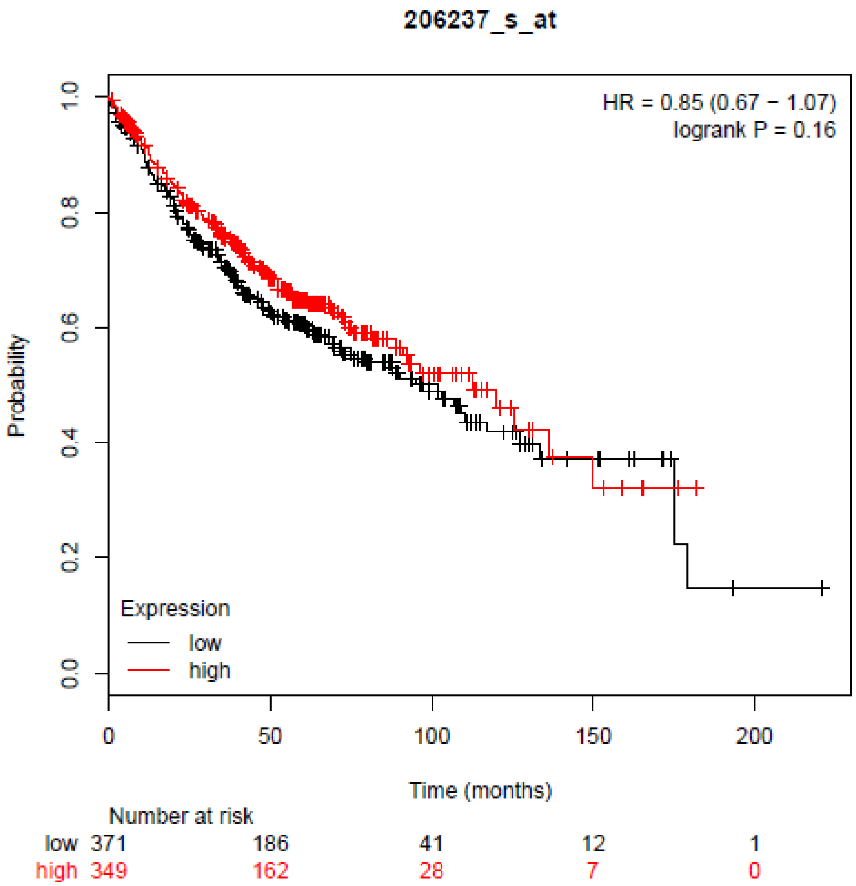

Kaplan-Meier curves of overall survival for patients with non-small adenocarcinoma of lung with low or high heregulin mRNA expression. Median values of heregulin mRNA is a cutoff value for dividing low (n = 362) and high (n = 358) expression groups. Curve and log-rank test was calculated and drawn by online Kaplan-Meier plotter.

Gyorffy B, Surowiak P, Budczies J, Lanczky A. Online survival analysis software to assess the prognostic value of biomarkers using transcriptomic data in non-small-cell lung cancer, PLoS One, 2013 Dec 18;8(12):e82241.
